# Empirical Mode Decomposition-Derived Entropy Features Are Beneficial to Distinguish Elderly People with a Falling History on a Force Plate Signal

**DOI:** 10.3390/e23040472

**Published:** 2021-04-16

**Authors:** Li-Wei Chou, Kang-Ming Chang, Yi-Chun Wei, Mei-Kuei Lu

**Affiliations:** 1 Department of Physical Medicine and Rehabilitation, China Medical University Hospital, Taichung City 40402, Taiwan; chouliwe@gmail.com; 2 Department of Physical Medicine and Rehabilitation, Asia University Hospital, Asia University, Taichung City 41354, Taiwan; 3 Department of Physical Therapy and Graduate Institute of Rehabilitation Science, China Medical University, Taichung City 40402, Taiwan; 4 Department of Medical Research, China Medical University Hospital, China Medical University, Taichung City 40402, Taiwan; 5 Department of Computer Science and Information Engineering, Asia University, Taichung City 41354, Taiwan; rex27938627@gmail.com; 6 Department of Digital Media Design, Asia University, Taichung City 41354, Taiwan; 7 Department of Early Childhood Education, Asia University, Taichung City 41354, Taiwan; mkuei2002@yahoo.com.tw

**Keywords:** fall risk, approximate entropy, sample entropy, empirical mode decomposition, intrinsic mode functions

## Abstract

Fall risk prediction is an important issue for the elderly. A center of pressure signal, derived from a force plate, is useful for the estimation of body calibration. However, it is still difficult to distinguish elderly people’s fall history by using a force plate signal. In this study, older adults with and without a history of falls were recruited to stand still for 60 s on a force plate. Forces in the x, y and z directions (Fx, Fy, and Fz) and center of pressure in the anteroposterior (COPx) and mediolateral directions (COPy) were derived. There were 49 subjects in the non-fall group, with an average age of 71.67 (standard derivation: 6.56). There were also 27 subjects in the fall group, with an average age of 70.66 (standard derivation: 6.38). Five signal series—forces in x, y, z (Fx, Fy, Fz), COPX, and COPy directions—were used. These five signals were further decomposed with empirical mode decomposition (EMD) with seven intrinsic mode functions. Time domain features (mean, standard derivation and coefficient of variations) and entropy features (approximate entropy and sample entropy) of the original signals and EMD-derived signals were extracted. Results showed that features extracted from the raw COP data did not differ significantly between the fall and non-fall groups. There were 10 features extracted using EMD, with significant differences observed among fall and non-fall groups. These included four features from COPx and two features from COPy, Fx and Fz.

## 1. Introduction

**Figure 1:**
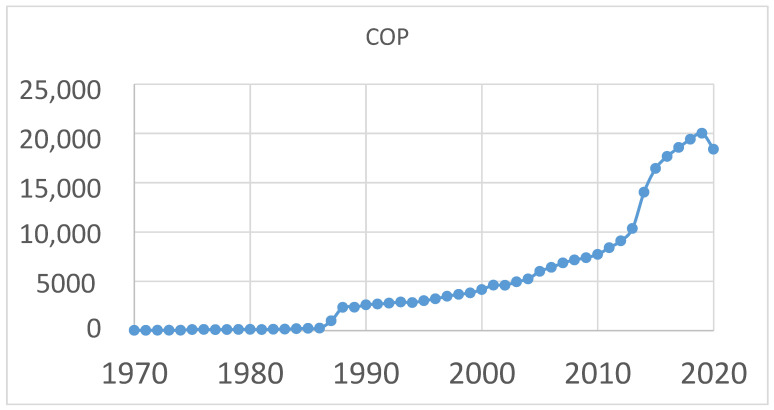
Academic papers related to center of pressure. The x-axis is publication years; the y-axis is the number of publications. Data are retrieved from PubMed.

The aging of society worldwide has raised awareness on topics associated with the health of older adults, particularly those relevant to preventive medicine [1]. Falls are both one of the most critical factors affecting the health of this population and the second most common cause of death after traffic incidents. Each year, 37.3 million cases of falls in Taiwan require medical treatment. Injuries caused by falls may be fatal, and older adults are particularly susceptible to severe injury or death from falls. In the United States, 20–30% of older adults sustain a moderate or severe injury from falls. Effective tools for exploring falls in older adults include wearable devices [2,3,4,5,6] and sociological questionnaires [7,8,9]. Balance, an effective biomechanical indicator for assessing the risk of falls, is mainly evaluated using center of pressure (COP), which is measured with a force plate. The force plate collects data from forces and moments along the anterior, posterior, leftward, rightward, upward and downward directions of the plate, as well as the combined moments along the three major axes. These data can then be used to determine the COP relative to the origin point of the force plate [10]. Applications and research of COP have received increased scholarly attention in recent years. Figure 1 presents a list of academic papers in which “center of pressure” was a keyword. Notably, data from the PubMed database indicate that the number of papers relevant to COP has gradually increased on an annual basis. COP signals are a set of time domain signals from which feature parameters can be determined. Various methods exist for extracting features from COP signals [11]. Time domain, frequency domain, time–frequency domain and nonlinear features in COP signals are commonly subjected to analysis [12,13].

Although the amplitude and standard deviation of time–frequency domain features are the most commonly examined statistics in COP analysis, entropy features have received a significant amount of attention due to the complexity of COP signals [13]. In a compilation of studies published in recent years, Kędziorek et al. concluded that sample entropy is the most commonly used tool for evaluating postural stability [14]. Sample entropy, which generates a unit-less number between 0 and 2, is used to measure signal patterns and complexity. Values of 0 and 2 denote body sway in a perfectly monotonous pattern and completely irregular and unpredictable swaying motions, respectively [15]. In a study by Ahmadi et al., sample entropy (as an indicator of COP) was higher in participants who were walking while playing games than in participants who were only walking [16]. Chao et al. used two nonlinear parameters, namely sample entropy and multivariate sample entropy, to compare the COP of individuals with regular feet and flat feet [17].

In empirical mode decomposition (EMD), an effective preprocessing method proposed by Norden E. Huang, a member of the United States National Academy of Engineering, data are decomposed into intrinsic mode functions (IMFs) [18] before undergoing the Hilbert–Huang transform, through which the instantaneous phase and frequency can be determined. This method is highly effective for processing nonhomeostatic and nonlinear signals because it entails multiple iterations of selection before IMF identification is complete. This selection process is similar to the concept of the filter bank. The initially calculated IMF has the highest frequency. Next, a band-pass-like filter is used to progressively decompose the signal into various frequencies until it exhibits the pattern of a sine wave. In contrast to band-pass filters with a fixed bandwidth, those decomposed using EMD vary with the characteristics of the input signal. The concept of EMD is similar to that of dynamic bandwidth filters. Meaningful signal components can be identified in the processing of physiological signals using EMD. This approach has been applied in the use of noise filters for noise suppression in electrocardiogram signals [19] and the analysis of signals acquired from electroencephalograms [20]. In general, IMF decomposition yields signals containing meaningful information in several IMFs, whereas the remaining IMFs contain noise only. Compared with the direct extraction of features from original signals, using EMD to decompose IMFs with varying signal components and then extracting features from the decomposed IMFs is more effective for the determination of differences between the extracted features. Moreover, this approach can enhance the outcomes of the subsequent signal classification process. EMD has been successfully applied to COP analysis in several studies [21,22,23,24].

The data investigated in this article are from an open database developed by Santos et al. [16]. Montesinos et al. analyzed the same dataset and explored the various default parameters of approximate entropy and sample entropy relevant to these features [17]. However, there are few entropy features that are statistically significant between fall and non-fall groups of elderly subjects. The hypothesis of this study is as follows:

**Hypothesis** **1.** *EMD-derived COP entropies will contribute more significant features distinguishing fall and non-fall groups of elderly subjects than the raw COP signal*.

In the present study, EMD was used to obtain multiple IMF combinations from COP data sourced from an open database. Next, the COP signals were subjected to linear and nonlinear feature extraction to differentiate between older adults with and without a history of falls. Finally, significant signal features were identified. 

## 2. Materials and Methods

### 2.1. Subject and Data Information

The present study used data on the 163 participants examined in the study by Santos et al. [25], in which data were collected using an amplifier (Optima Signal Conditioner; AMTI, Watertown, MA, USA) and a force platform (OPT400600-1000; AMTI) paved with a foam block of 6 cm in height (Balance Pad; Airex AG, Sins, Switzerland). The sampling frequency was 100 Hz, and the sampled data were smoothed using a 10 Hz fourth-order zero-lag low-pass Butterworth filter. Next, the data were processed using NetForce software (Version 3.5.3; AMTI) to determine the forces and moments of forces of the platform (Fx, Fy, Fz, Mx, My and Mz). Participants aged over 65 years were further divided into fall and non-fall groups. Specifically, those who had experienced falls in the past 12 months or received a high score on the Short Falls Efficacy Scale International were assigned to the fall group. The demographic characteristics of the two groups are shown in Table 1. The fall and non-fall groups comprised 49 and 27 participants, respectively. The data consisted of five signal series, namely the forces in the x, y and z directions (i.e., Fx, Fy, and Fz) and COP in the anteroposterior (COPx) and mediolateral directions (COPy). Signal features were then extracted from the data.

### 2.2. EMD

The EMD process is described as follows, using the x(t) signal as an example [19].

Step 1: Label the maximum and minimum values of the x(t) signal;

Step 2: Connect the extrema to form the upper and lower envelopes;

Step 3: Determine the mean functions, or m(t), of the upper and lower envelopes;

Step 4: Solve the equation d(t) = x(t) − m(t);

Step 5: If d(t) is a zero-mean process, the calculation is terminated, and d(t) becomes the first IMF (IMF1). Otherwise, x(t) is replaced with d(t), and the calculation process is repeated from step 1;

Step 6: Residue signal r(t) = x(t) − IMF1(t);

Step 7: x(t) is replaced with r(t), and steps 1–6 are repeated to determine the second IMF, or IMF2(t). These procedures are repeated for n iterations to obtain IMFn(t). The iteration is terminated when r(t) becomes a monotonic function. Accordingly, the original data are decomposed into n IFMs, an IMFn(t) and a residual signal function r(t).

R was used as the programming language in the present study. To execute the EMD algorithm, an R package for EMD was downloaded and then input into the library (EMD). The command “emd” was then used to perform the calculations for the determination of IMF1–IMF7. After IMF7 was derived, the data signals exhibited a sine wave pattern, indicating that they no longer contained any information relevant to COP. Accordingly, the decomposition process was terminated. Five signal series—namely Fx, Fy, Fz, COPX and COPy—were used, with each data series being decomposed into seven IMFs.

**Figure 2:**
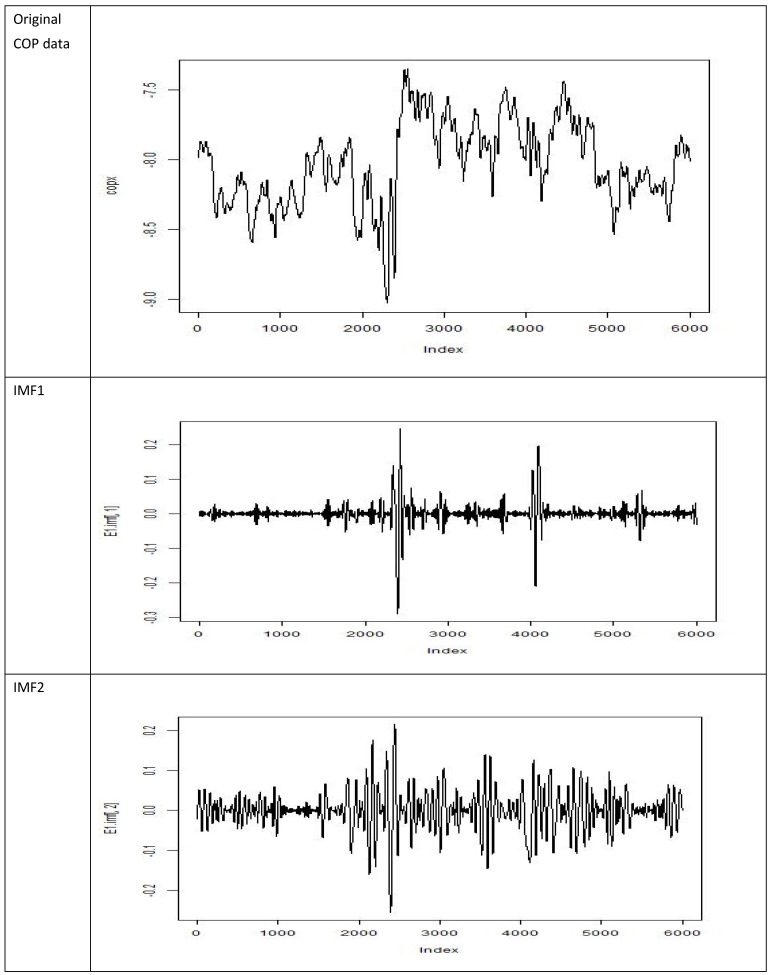

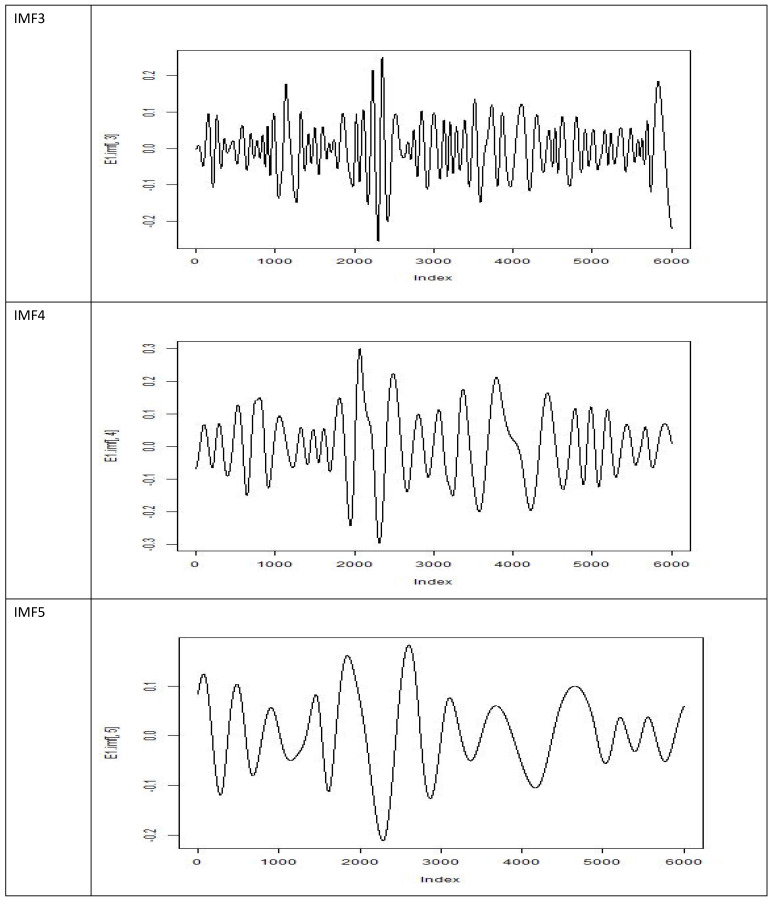

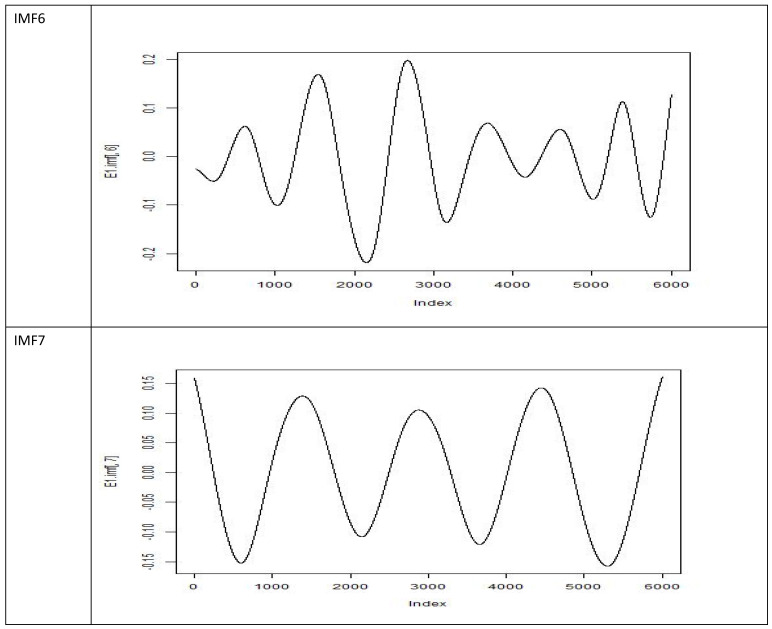
Center of pressure (COP) data and related intrinsic mode function (IMF) decompositions.

The present study used COP measurement to differentiate between older adults with and without a history of falls. Adequate feature parameters with significant between-group differences were identified. This was achieved by preprocessing COP time series signals. In the study by Santos et al. [25], COP signals were acquired by using a sampling frequency and sampling time of 100 Hz and 60 s, respectively, and a seventh-order IMF decomposition process was applied to the first batch of signals (No. BDS00001). Figure 2 presents the original COP data and the resulting IMFs (IMFs 1–7). As the IMF was progressively decomposed, signals with low-frequency components were obtained. IMF1 contained signals with the highest frequencies. 

### 2.3. COP Feature Extraction and Statistics

After the Fx, Fy, Fz, COPx and COPy data of each participant were subjected to EMD, the time domain, frequency domain and nonlinear features of each IMF were determined. Information on each feature is presented in Table 2. Time domain feature definitions are represented in the following equations: 

Mean = mean(abs(signal(t)));

STD = sd(signal(t));

Coefficient of variation (CV) = STD/Mean;

The feature extraction code is attached in Appendix A.

**Figure 3:**
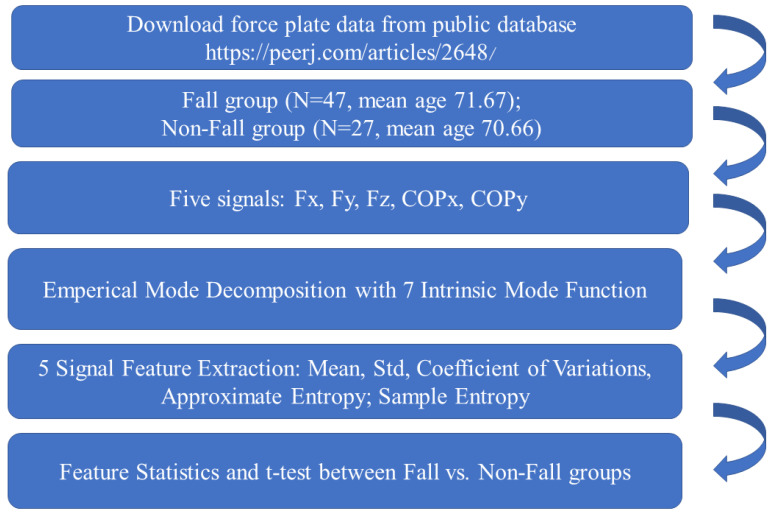
Experiment flowchart.

A total of eight time series (the raw data and IMF1–IMF7) were determined from each signal source, and five features (i.e., three time domain features and two nonlinear features) were extracted from each time series, yielding a total of 200 features (5 × 8 × 5). The features acronym is defined as signal source_IMF level_Features. There are five signal sources—Fx, IFy, Fz, COPx and COPy—and there are seven IMF levels. For example, COPx_0_ApEn is approximate entropy derived from COPx. Fz_7_STD is the standard derivation derived from the seventh IMF of the Fx signal. The features were categorized by group (fall vs. nonfall), and the means and standard deviations for each group were calculated accordingly. Next, *t* tests were performed to evaluate the between-group differences. The alpha value for statistical significance was set as 0.05. Figure 3 presents the complete experimental process.

## 3. Results

Appendix B presents the means, standard deviations and t test results of the 200 features. Table 3 (A,B) lists the features with significant differences. The features extracted from the raw COP data did not differ significantly between the fall and non-fall groups (Table 3 (A)). This finding is consistent with that reported by Montesinos et al. [26]. By contrast, significant differences were observed among 10 features extracted using EMD (Table 3 (B)). Specifically, four features from COPx (3_ApEn, 4_ApEn, 3_ sample entropy, and 4_ sample entropy), COPy (5_mean, 5_STD), Fx (1_ApEn, and 1_sample entropy) and Fz (7_mean, 7_STD) were significant, respectively. None of the features from Fy differed significantly.

## 4. Discussion

The results indicate that features extracted from the functions decomposed through EMD can be used to distinguish between older adults with and without a history of falls. Montesinos et al. attempted to use entropy features to analyze COP data [26]. However, this approach can only reveal significant differences between young and elderly participants. To the best of the authors’ knowledge, the present study is the first to use entropy features, EMD and COP signals to identify older adults at risk of falls. In contrast to Montesinos et al. [26], the present study did not further explore the effect of default parameters, namely time series length, subseries length and tolerance. However, this does not affect the objectives, which are centered on the integrated use of EMD and entropy features, or the findings.

Why can the features derived from IMF make statistical differences between fall/non-fall groups more successfully than raw COP data? The characteristics of EMD are similar to those of a continuous band-pass filter with varying bandwidths; IMFs with lower values exhibit higher frequencies. In general, COP signals among older adults with and without a history of falls have common components that can be identified using EMD. In the present study, the significantly different features in the COPx signal series originated from IMF3 and IMF4, which corresponded to signals in the middle frequency band. By coincidence, these significant features were also nonlinear. The mean sample entropy of the fall and non-fall groups in IMF3 was 0.10 and 0.12. In IMF4, it was 0.05 and 0.06. In short, sample entropy was lower in the fall group than in the non-fall group. As mentioned, higher sample entropy values represent more irregular and unpredictable swaying motions. In the present study, the x-axis corresponded to the anteroposterior direction. Because the fall group displayed swaying motions of a greater magnitude in this direction, their sample entropy was lower, thereby increasing the risk of falls. 

The present findings serve as a reference for the early detection of fall risk in older adults. Specifically, this can be achieved by instructing older adults to stand on a force plate for 1 min and by analyzing the entropy calculated from EMD-derived IMFs. Fall prevention programs can then be implemented for the individuals at higher risk of falls. The examination of the entropy features not discussed in the present study can be used to confirm the present findings. Moreover, effective algorithms can be tested to simplify and accelerate the entropy calculation process. In addition, artificial intelligence classifiers can be integrated to classify and identify fall risk in older adults.

## 5. Conclusions

In this article, raw data measured from a force plate were investigated. There was no significant difference for features derived from raw data to distinguish elderly subjects as fall and non-fall groups. After applying the EMD algorithm to raw data, there were 12 features extracted; using EMD, significant differences were then observed between fall and non-fall groups. These were four features from COPx (3_ApEn, 4_ApEn, 3_sample entropy, and 4_sample entropy) and two features from COPy (5_mean, 5_STD), Fx (1_ApEn, and 1_sample entropy) and Fz (7_mean, 7_STD). Therefore, EMD is useful to extract information from the COP to detect fall risk.

## Figures and Tables

**Table 1:** Demographic characteristics of the participants. ^a^ The illnesses were hypertension, hypercholesterolemia and glaucoma. ^b^ The disabilities were categorized as hearing, physical and visual impairments. ^c^ The options for the number of medications were 0, 1 to 2, 3 to 4 and ≥5.

	Non-Fall Group	Fall
original subject number	49	27
Subject included	49	27
Gender	F35/M14	F25/M2
Age (mean, SD)	71.67 (6.56)	70.66 (6.38)
Illness Yes#/No ^a^	Yes (*n* = 43) No (*n* = 6)	Yes (*n* = 24) No (*n* = 3)
Disability ^b^	Yes (*n* = 6) No (*n* = 43)	Yes (*n* = 6) No (*n* = 21)
Medication ^c^	[4;24;18;3]	[5;9;9;4]

**Table 2:** COP feature definitions. EMD: empirical mode decomposition.

Type	Content
Raw data for each trial	Fx, Fy, Fx, COPx, COPy
EMD decomposition	IMF1 to IMF7
Input signal sequence	Raw data and derived IMF1 to IMF7
Time domain features	Mean, standard derivation, coefficient of variation (CV)
Nonlinear features	Approximate entropy; sample entropy

**Table 3:** Means and standard deviations in the t test results of the fall and non-fall groups. * *p* < 0.05; ** *p* < 0.01.

**(A) Raw COP Signal Features**	**Fall**	**Non-Fall**	***p* Value**
COPx_0_mean	−1.175 (2.412)	−1.072 (1.855)	0.849
COPx_0_CV	−0.934 (3.557)	−0.268 (1.539)	0.260
COPy_0_mean	0.035 (0.946)	0.154 (0.993)	0.656
COPy_0_CV	0.016 (1.222)	0.574 (2.081)	0.284
COPx_0_sample entropy	0.628 (0.226)	0.667 (0.196)	0.486
COPy_0_sample entropy	0.586 (0.163)	0.619 (0.189)	0.511
COPx_0_ApEn	0.486 (0.094)	0.516 (0.068)	0.138
COPy_0_ApEn	0.470 (0.057)	0.494 (0.079)	0.23
**(B) EMD Derived Features**	**Fall**	**Non-Fall**	***p*** **Value**
COPy_5_mean	0.076 (0.039)	0.058 (0.029)	0.029 *
Fz_7_mean	0.039 (0.020)	0.029 (0.012)	0.015 *
COPy_5_STD	0.093 (0.049)	0.072 (0.036)	0.032 *
Fz_7_STD	0.048 (0.026)	0.037 (0.015)	0.017 *
COPx_3_ApEn	0.117 (0.029)	0.137 (0.030)	0.007 **
COPx_4_ApEn	0.056 (0.010)	0.062 (0.011)	0.019 *
FX_1_ApEn	0.609 (0.063)	0.633 (0.019)	0.016 *
COPx_3_sample entropy	0.107 (0.030)	0.125 (0.028)	0.013 *
COPx_4_sample entropy	0.055 (0.011)	0.061 (0.011)	0.037 *
FX_1_sample entropy	0.547 (0.096)	0.581 (0.031)	0.023 *

## Data Availability

Extracted data features are listed at the following link: https://github.com/loveso1G/C02.git (accessed on 6 March 2020).

## Appendix A

The R code used in this article can be found at the following link: https://github.com/loveso1G/code.git (accessed on 6 March 2020).

## Appendix B

The detailed information for the means, standard deviations and t-test results of all features between fall and non-fall groups is listed in a file at the following link: https://github.com/loveso1G/C02.git (accessed on 6 March 2020).
